# PaPrBaG: A machine learning approach for the detection of novel pathogens from NGS data

**DOI:** 10.1038/srep39194

**Published:** 2017-01-04

**Authors:** Carlus Deneke, Robert Rentzsch, Bernhard Y. Renard

**Affiliations:** 1 Research Group Bioinformatics (NG4), Robert Koch Institute, 13353, Berlin, Germany

## Abstract

The reliable detection of novel bacterial pathogens from next-generation sequencing data is a key challenge for microbial diagnostics. Current computational tools usually rely on sequence similarity and often fail to detect novel species when closely related genomes are unavailable or missing from the reference database. Here we present the machine learning based approach PaPrBaG (Pathogenicity Prediction for Bacterial Genomes). PaPrBaG overcomes genetic divergence by training on a wide range of species with known pathogenicity phenotype. To that end we compiled a comprehensive list of pathogenic and non-pathogenic bacteria with human host, using various genome metadata in conjunction with a rule-based protocol. A detailed comparative study reveals that PaPrBaG has several advantages over sequence similarity approaches. Most importantly, it always provides a prediction whereas other approaches discard a large number of sequencing reads with low similarity to currently known reference genomes. Furthermore, PaPrBaG remains reliable even at very low genomic coverages. CombiningPaPrBaG with existing approaches further improves prediction results.

The vast amount and diversity of bacteria on Earth, together with ever increasing human exposure^1^, suggests that we will be continuously confronted with novel bacterial pathogens, too. Encouragingly, next-generation sequencing (NGS) has emerged as a novel, powerful diagnostic tool in this regard. However, the direct NGS-based characterisation of novel pathogenic strains or even species is still problematic when closely related genomes are unavailable or missing from the respective reference database. Here we introduce a machine learning based approach, PaPrBaG, which overcomes genetic divergence in predicting bacterial pathogenicity by training on a wide range of species with known pathogenicity phenotype. Importantly, even if this is avoided for practical reasons at some points throughout this (and related) work, one may more cautiously speak of *pathogenic potential* than pathogenicity, given that the latter is ultimately governed by the complex interplay between host (state) and pathogen.

## Existing Methods

Existing approaches amenable to pathogenicity prediction broadly fall into two classes: protein content based and whole-genome based. Where assembled genomes are available, the presence/absence pattern of certain protein families can be expected to correlate with complex phenotypes, e.g. pathogenicity. This is primarily based on the presence of virulence factors (VFs) – often acquired through horizontal gene transfer^2^ – or the absence of more common genes (functions) that become dispensable when e.g. host-specific pathogens evolve from commensal ancestors^3^. Three recent studies rely on these considerations.

The BacFier method by Iraola *et al*.^4^ was the first to apply the described approach on a large scale. The authors defined eight VF categories and obtained 814 related VF protein families from KEGG^5^. They further used a set of 848 human-pathogenic (HP) and generally non-pathogenic genomes broadly covering bacterial taxonomy. Using a support vector machine (SVM) based approach, they subsequently selected the most discriminative subset of all 814 features (presence/absence of a given family in a given genome) through cross-validation. For PathogenFinder, Cosentino and coworkers^6^ compiled a list of 1,334 genomes with available pathogenicity information. Of those, 885 had been published before November 2010 and formed the training set. Their proteins were clustered into 28,000 families using CD-HIT^7^. Families significantly enriched in either HPs or non-HPs were assigned a signed weight value depending on the degree of enrichment. The 449 genomes published later formed the test set. Their phenotype was predicted by assigning the encoded proteins to the previously generated families and summing up the associated weights, respectively. In a more focused and qualitative study, Barbosa and colleagues^8^ used a manually labelled set of 240 actinobacterial genomes and identified just under 30,000 protein families using their own Transitivity Clustering method^9^. The authors further distinguished between HPs, broad-spectrum animal pathogens, opportunistic HPs and non-pathogens.

Pathogenicity may also be predicted using a range of established tools that were originally developed for mapping NGS reads to reference genomes and/or classifying them taxonomically. In doing so, the likelihood of pathogenicity increases with proximity to a pathogenic reference. While the challenge of detecting known pathogens in mixed (e.g. clinical) samples is very much related to general metagenomic analysis workflows^10^,^11^,^12^, such have rarely been used for predicting the presence of novel pathogens. While classic alignment tools like BLAST^13^ may be used for mapping with high sensitivity but relatively low throughput, the opposite applies to dedicated read mappers such as Bowtie2^14^ and BWA^15^, whose performance deteriorates quickly for highly divergent query strains or even novel species. Still, the latter are widely used in non-predictive NGS analysis pipelines. PathoScope^16^,^17^, for example, provides a statistical filtering scheme to resolve mapping conflicts, i.e. reads mapping to different references. Clinical PathoScope^18^ is particularly useful to remove large numbers of contaminant reads, e.g. human ones in the context of clinical samples. Instead of using existing mappers, the SURPI pipeline^19^ relies on two custom-built tools for nucleotide and BLASTX-like translated alignment. Both are shown to scale better with data set size when compared with their conventional competitors whilst maintaining similar performance. The translated alignment step with *de-novo* assembled contigs is reported to increase sensitivity particularly in the viral domain.

Composition-based methods compare the distributions of different compositional features (usually k-mer occurrence or frequency) in the query and reference sequences. Kraken^20^, for example, tries to match 31-mers found in the query to a precomputed database, which maps them to the lowest common ancestor taxon of all reference genomes they occur in. Similarly, MetaPhlAn^21^ first creates a compact database of clade-specific marker genes (ranging from strain to phylum level), which it then uses for prediction. NBC^22^,^23^ calculates k-mer frequency profiles of all references and uses them to train a naïve Bayesian classifier. Other machine learning approaches use kernelised nearest neighbour^24^ or hierarchical structured-output SVMs^25^,^26^. Hogan *et al*.^27^ trained binary classifiers on two reference groups (e.g. two phyla or one vs. all others). In this case, a database of ‘competing’ classifiers must be built for wide taxonomic coverage.

## Motivation and Aims

The existing approaches outlined above have different strengths and weaknesses depending on the specific usage scenario. Here, the scenario is the robust and user-friendly prediction of pathogenic potential based on raw NGS data from newly discovered bacterial strains or species with potentially large sequence divergence. Note that the latter is not an uncommon event: e.g., Schlaberg and colleagues^28^ identified 673 isolates belonging to undescribed species. More recently, sequencing of bacterial isolates from patients in an intensive care unit led to the discovery of 428 potentially novel species within a single year^29^.

While protein content based methods show great potential for not only prediction but also qualitative analyses (e.g. pinpointing clade-specific VFs), and even for identifying yet uncharacterised VFs (as illustrated in the above-cited publications), their primary, shared drawback is the dependence on genome assembly and annotation. These steps are both time-consuming and, particularly in novel-species and/or low-coverage scenarios, error-prone. Further, these methods neglect the signal potentially found outside of protein-coding genes. While dedicated read mappers do not share these problems, they may still struggle with highly divergent strains or (even) novel species; in turn, this impacts frameworks like PathoScope. The same applies to methods depending on long, gapless k-mer matches, like Kraken and NBC. BLAST, on the other hand, is generally considered too slow for large-scale read mapping. Finally, we are not aware of any previous studies using a read-based machine learning approach for pathogenicity prediction, in conjunction with a comprehensive evaluation. Given our above-stated scenario, however, more fundamental differences exist between these genome-based methods and what we were aiming for. They are (i) heavily influenced by the taxonomic coverage of the underlying data sets, (ii) make taxonomic instead of phenotypical predictions, and (iii) are not designed to make predictions per se (but rather identify already known organisms). In summary, while all these methods are highly useful in different contexts, they do not necessarily fit the task at hand. Therefore, we developed PaPrBaG: Pathogenicity Prediction for Bacterial Genomes.

In an important preliminary step, we compiled a comprehensive set of genomic data and metadata. Based on the latter, we then established a system of rules to automatically identify HP and non-HP bacteria. For the prediction task, we introduced several new compositional features and used them for training as well as querying a binary random forest classifier. These are known to be fast, error-tolerant and capable of dealing with a large number of features. Finally, we provide a solid evaluation, also comparing against other types of methods. This may serve as a guideline for users to select the most appropriate method for a given task, e.g. in clinical settings. A user-friendly R package is additionally provided at https://github.com/crarlus/paprbag.

## Data

No comprehensive standard resource listing bacterial strains with or without pathogenic potential in human is publicly available. However, the Integrated Microbial Genomes (IMG) system collects a wide range of metadata on microbial genome projects^30^. We accessed the IMG web site on 04/06/2015 and downloaded a table containing all available data. In a first step we pre-filtered the IMG data for the key *Bacteria* in the field *Domain*, for *Finished* or *Permanent draft* in *Status* and for *Genome Analysis* in *Project Type*; the latter serves to exclude metagenomic studies. We furthermore excluded any genomes for species marked as *unclassified*.

To infer the pathogen label we search for entries that contain the term *Pathogen* in the fields *Phenotype* or *Relevance*. Additionally, all genomes that contain an entry in the field *Diseases* are labelled as pathogenic. We inferred non-pathogens by searching for the keyword *Non-pathogen* in the field *Phenotype*. Note that no further field clearly designates non-pathogens. In particular, from a missing entry in the field *Disease*, it does not follow that the organism is not a pathogen. The same holds for (reference) genomes that were sequenced as part of the Human Microbiome Project (HMP), since those include both pathogens and commensals^31^,^32^. If contradicting rules were met for an entry, e.g. non-pathogenic phenotype yet still an annotated disease, the label ‘unknown’ was assigned and the genome excluded from further analysis.

For the present study, and with a clinical setting in mind, we were interested in HPs and non-HPs only. Therefore, including e.g. plant pathogens or non-pathogenic soil bacteria could result in misleading conclusions (those could be used in analogous studies for other habitats and hosts, though). Bacteria with human host were identified using the following set of rules: either the entries *human* or *Homo Sapiens* are found in the fields *Host Name, Ecosystem Category* or *Habitat*; or the field *Study Name* contains the entry *HMP*. For further analysis we kept all entries with human host and either pathogenic or non-pathogenic phenotype.

We finally obtained labels for 2,836 bacterial strains (177 non-HPs and 2,659 HPs). These belong to 422 different species. On the species level, we found 363 pure HPs and 53 pure non-HPs. For only 6 species, we found that labels were mixed between strains. Most strikingly, *Escherichia coli* comprises 172 HP and 93 non-HP strains. For five other species, *Campylobacter jejuni, Listeria monocytogenes, Staphylococcus epidermidis, Peptoclostridium difficile* and *Clostridium botulinum*, we found one or two non-HP strains vs. many HP strains. However, these species are commonly known as HPs and therefore the non-HP entries were excluded from further analysis. Interestingly, even on the genus level the pathogenicity phenotype is highly conserved in our data set. Only 13 out of 135 genera contain both HP and non-HP species: *Bacteroides, Campylobacter, Clostridium, Escherichia, Haemophilus, Lachnoclostridium, Listeria, Neisseria, Parabacteroides, Peptoclostridium, Staphylococcus, Streptococcus* and *Tannerella*. Further, 46 of the remaining 122 contain only a single species each.

The resulting table of label data is provided in the supplied R package. The genomic sequence FASTA files were retrieved by querying NCBI Entrez^33^ with the corresponding *BioProject accessions* as provided in the IMG table (on August 24, 2015).

## Methods

### Approach

**Figure 1:**
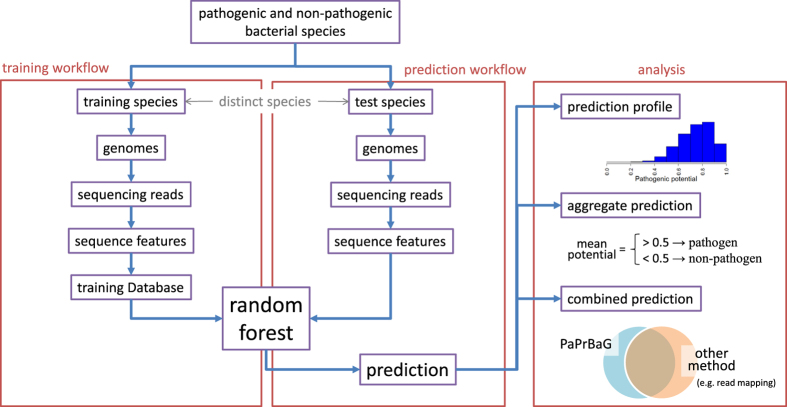
Overview of PaPrBaG workflow. Reads are simulated from genomes in both the training (left) and prediction (center) workflows, from which features are extracted. The training sequence features together with the associated phenotype labels compose the training database, on which the random forest algorithm trains a pathogenicity classifier. This classifier predicts the pathogenic potential for each read in the test set. From these raw results, the prediction profile, the genome aggregate prediction and a combined prediction can be generated (right).

Figure 1 summarises the individual steps of PaPrBaG. The supervised machine learning setup comprises a training and a prediction workflow. The entire set of HP and non-HP bacterial species is divided into non-overlapping training and test sets. Subsequently, selected genomes from all species are fragmented into reads (see subsection *Benchmark*), from which a range of sequence features are extracted (subsection *Features*). The training sequence features together with the associated phenotype labels compose the training database, on which the random forest algorithm trains a pathogenicity classifier (subsection *Machine Learning*). In turn, this classifier predicts the pathogenic potential for each read in the test set. Based on these raw results various analysis steps can be performed. This section further provides a summary of the different benchmark approaches (subsection *Benchmark*), evaluation strategies (subsection *Metrics*) and metagenome configuration (subsection *Metagenomic example*) used in the Results section.

### Machine Learning

A random forest classifier^34^ was trained using the below-described features and (genome) pathogenicity labels for each read in the training data set. We chose this classifier type because it combines high accuracy, fast prediction speed and the capability to deal with noisy data^35^,^36^. Among the different implementations of the random forest algorithm available, we opted for *ranger*^37^,^38^ since it is one of the fastest - implemented in C^++^ — and can handle large data sets. We used probability forests, which return the fraction of votes for each class. This can also be interpreted as the prediction probability. We therefore refer to the prediction probability of the HP class as the *pathogenic potential* of a read.

As another advantage, the random forest approach has only few tuneable parameters. In this context, we found it sufficient to train 100 trees per forest, as more trees do not lead to better predictions (see Supplementary Fig. S1). Fluctuations play some role for small tree numbers between 20 and 50 trees per forest. Forests with 100 trees or more, however, yield very similar per-read predictions and nearly identical per-genome predictions, justifying our parameter setting. We further adjusted the minimum size for terminal nodes. High numbers can result in impure terminal nodes and smaller trees. Changing it from 1 (default and our choice here) to 10 had no effect, while sizes above 1,000 led to overfitting. Changing any further parameters had no substantial effect, respectively. The trained random forest objects are available on github.

### Features

For the machine learning task, a set of informative features must be extracted from the read sequences. We implemented a number of different feature types to capture the information content present in a sequencing read.

#### Genomic features

Different features were extracted from DNA sequences, all of which based on k-mer occurrence patterns. Since we analyse read data, strand information is not available. Therefore, we cast all features symmetrically so that the occurrences of a word and its reverse-complement were considered jointly, a common strategy in related methods^39^,^40^.

A first features type is the relative k-mer frequency. We found that including monomers, dimers, trimers and tetramers led to good results, but higher values of k did not lead to further improvement. The occurrences of longer k-mers are less likely to overlap among highly divergent sequences. Conversely, the consideration of a large number of uninformative long k-mers can compromise prediction performance. However, as focussing on selected longer sequence motifs can still be beneficial for classification, we also recorded the frequencies of the 100 most abundant 8-mers in an independent set of bacterial genomes, scanning both strands and allowing for one mismatch. *Spaced words* were introduced for the alignment of dissimilar sequences^41^,^42^. Thus, their incorporation is useful in the context of novel species discovery. Spaced words denote the occurrence of all k-mers interrupted by *(l-k)* spacers in a word of length *l*. For this analysis we searched for all symmetric 4-mers in a spaced word of length 6.

#### Protein features

Bacterial genomes are known to be densely packed with proteins^43^. Since protein sequences are evolutionarily more conserved than DNA sequences, peptide features can provide additional valuable information. A read might (partially) cover a protein sequence, but the correct reading frame is unknown. However, longer DNA sequences tend to contain frequent stop codons in the anti-sense frames by chance. Therefore, as a simple heuristic, we generally used the frame and strand with the fewest number of stop codons. This frame was translated into a peptide sequence and several types of features were extracted: codon frequencies, relative monopeptide and dipeptide frequencies, amino acid properties and Amino Acid Index (AAIndex)^44^,^45^,^46^ statistics. The amino acid property features consist of the relative frequencies of tiny, small, aliphatic, aromatic, non-polar, polar, charged, basic and acidic residues^47^. Finally, the AAIndex assigns scores for diverse properties (often based on peptide secondary structure) to each residue. From 544 indices, we selected the 32 with the lowest pairwise correlation. Features were obtained by computing the product of the amino acid frequencies and their associated index scores. In total, we included 948 features in the classification workflow.

#### Feature importance

As measured by both the permutation and Gini tests, the most important features come from the DNA monomer, dimer and trimer feature groups (see Supplementary Table S1). Among the 100 most important features, the tetramer, codon frequency, AAIndex and spaced words groups are also prevalent. We estimated the importance of the different groups by searching for the highest scoring member of each group. The resulting order of group importance was trimer, monomer, dimer, tetramer, spaced words, AAIndex score, codon frequencies, monopeptides, DNA motifs, amino acid properties and dipeptides. Particularly the last five groups were of minor importance for the classification task.

### Benchmarking

#### Cross-validation strategy

To evaluate the developed classifier, we performed a five-fold cross-validation study. In this, we randomly distributed the HP and non-HP genomes into five equally-sized, non-overlapping parts, respectively. Further, only a single, randomly selected strain (genome) per species was considered. The significant variation in the number of strains per species (1 to over 200), which is mainly due to a pathogen study bias, would have otherwise translated into largely skewed training databases. Apart from markedly reducing label imbalance (the ratio decreases from 16 to 7), this also reduced the training data size. Further, the described approach reflects the scope of this work, which is predicting phenotypes on the species level. Still, for comparison, we included all strains of each training species in a separate benchmark study (see Results). *E. coli* played a unique role in that it possesses a large number of known HP and non-HP strains. In this study, we considered the HP strains only. Since the aim was to provide species-level predictions, the rationale was to be more sensitive towards pathogens.

#### Read simulation

For both the training and test data sets, we simulated 250 bp long Illumina reads. To that end we used the Mason read simulator with the default Illumina error model^48^. The number of reads sampled per genome differed for the training and test sets. In training binary classifiers, it is generally advantageous to show the learner an equal number of examples for both classes. Therefore, despite an HP to non-HP ratio of about 7:1, we decided to sample the same total number of reads per class, 10^6^. This represents a trade-off between genome coverage and training data size. An increase to 10^7^ reads did not substantially improve the prediction results. Further, the number of reads per genome in each class was chosen such that each genome had the same coverage, i.e. proportional to the size of the genome. Conversely, for the test data sets, we chose to sample up to a coverage of approximately 1 for each genome. The read simulation was repeated for each fold.

#### Comparison with other methods

We further compared the performance of PaPrBaG with a range of other tools, most of which were originally developed for taxonomic classification. First, we used Bowtie2, one of the commonly used read mappers combining speed and accuracy^49^. Further, we considered Pathoscope2^17^ as a dedicated pipeline for pathogen identification. More sensitive mapping is expected from BLAST^13^, which is still widely used in NGS pipelines. As a method integrating both alignment- and composition-based characteristics, we further assessed Kraken, which has emerged as one of the primary taxonomic classification tools^20^. Finally, we considered NBC as a composition-based machine learning method^22^,^23^. Unlike similar approaches, it allows the construction of a custom training database. We evaluated the performance of these tools using the PaPrBaG training and test genome sets, again using five-fold cross-validation.

#### Bowtie2

For read mapping, we used Bowtie2 (v2.2.4) in the *very-sensitive* configuration, which is highly tolerant towards mismatches and gaps. We obtained the 50 top alignments of each read. Parsing the resulting SAM file, we matched the best-scoring mapping of each read with the label database. When more than one alignment shared the best score, we chose a match to an HP over a match to a non-HP. For unmapped reads, no prediction could be made. Additionally, we repeated this mapping workflow for a larger reference genome set including all strains of the training species.

#### Pathoscope2

Pathoscope2 (v2.0.6) works as a post-mapping filter. Hence, we ran its ‘Identification’ module on the SAM file produced by Bowtie2. The resulting filtered SAM file was analysed as above to obtain label predictions. Also this analysis was repeated using the all-strain reference set.

#### Kraken

We provided Kraken (v0.10.5) with the training genome sequences, from which it builds a database based on clade-specific 31-mers. Based on this, the tool tries to classify each read taxonomically. The resulting species were matched to the label database via their NCBI taxonomy ID. If that failed, the species name (obtained via Kraken’s translation module) sometimes still produced a match. When Kraken’s prediction was not at species resolution, no prediction was made. Further, since matching 31-mers to divergent sequences may prove challenging, we repeated the entire analysis using 16-mers (Kraken-16). Note that the former shares characteristics of an alignment-based method, while the latter can be considered composition-based.

#### BLAST

We ran NCBI BLASTN (v2.2.28) with the option ‘-task dc-megaBLAST’, which is tailored for inter-species comparisons. Additionally, we chose an E-value threshold of 10. From the resulting BLAST output, the highest-scoring target was matched to the label database.

#### Naïve Bayes Classifier

We created a set of NBC (v1.0) training databases with word length 15 and then scored all test read sets against all training databases. For each read, we selected the highest-scoring hit and matched the species name to the label database. Since classification with NBC took very long, we had to use parallel threads.

### Evaluation metrics

#### Majority prediction rule

All tested methods yield predictions for individual reads, but ultimately we were interested in a single, integrated prediction for each genome. An individual read matching to an HP genome cannot, by itself, be deemed significant, given that also non-HP genomes will contain stretches similar to HP genomes. In PaPrBaG, we therefore average over all read-based prediction probabilities. If this value exceeds 0.5, the sample is classified as pathogenic. Likewise, for the other methods, we assign the class with the higher number of reads. This evaluation metric will henceforth be referred to as the *majority prediction rule*.

#### Minimum detection threshold

The *majority prediction rule* allows for a simple estimation of the pathogenic potential of a sample; however, it ignores uncertainty due to missing predictions. Therefore, we implemented a complementary metric, the *minimum detection threshold*. Here, a user can define the minimum fraction of reads that should be required for a confident phenotype prediction. As before, for a given test genome, we collect the read-based evidence. Then, a phenotype is considered for prediction only if the number of reads supporting it exceeds the minimum detection threshold. If both phenotypes are supported, the better-supported one determines the prediction. Further, no prediction is made when neither phenotype is sufficiently supported.

For both phenotypes, we assessed the fraction of correct predictions, which corresponds to the true positive rate (TPR) and true negative rate (TNR), respectively. We then summarised the performance using *informedness*, also known as Youden’s J statistic, which is a joint measure of specificity and sensitivity^50^. Formally, informedness is defined as *I* = TPR +* *TNR − 1 and ranges from −1 (only wrong predictions) to 1 (only correct predictions).

#### Consensus filter

Individual approaches may yield heterogeneous predictions, which makes it attractive to combine them to enhance prediction confidence. We therefore define a *consensus filter* as follows. First, we evaluate which predictions coincide between two methods. We then keep only the consensus subset for further performance evaluation.

#### Prediction certainty

Each prediction made by the *majority prediction rule* is associated with uncertainty. We defined the prediction certainty as |*μ* − 0.5| × 2, where *μ* denotes the majority prediction as discussed above. The result is a relative certainty value that always ranges from 0 (maximally uncertain) to 1 (maximally certain). Note that this value does not reflect the predicted class. We further normalised the certainty of each predictor by the highest certainty it reports for any genome. This is not a necessary step but aids visualisation.

### Metagenomic example

To illustrate the application of PaPrBaG in a metagenomic context, we simulated an artificial set of reads belonging to a set of known non-HPs (present in a given training fold) and a single unknown HP (not present in that fold). As HP we chose *Gardnerella vaginalis*, currently the only known representative of its genus. Since it is known to invade the female urogenital system, we then selected a range of non-HPs that are known to colonise the same habitat in healthy humans according to the HMP. These were *Lactobacillus salivarius, Lactobacillus reuteri, Lactobacillus rhamnosus, Bifidobacterium breve* and *Haemophilus parainfluenzae*, all of which found in the relevant training fold. Our further strategy resembled that used in ref. 20. Mimicking a clinical sample, where non-HPs (e.g. commensals) will generally vastly outnumber the given HP, we sampled 200,000 reads for each of the latter but only 6,604 reads (equivalent to a coverage of 1) for the former. In this controlled scenario we can identify the number of true positives (*G. vaginalis* reads mapped to another HP), false negatives (*G. vaginalis* reads mapped to a non-HP), and false positives (non-HP reads mapped to an HP).

## Results

In the following, we discuss the results of a five-fold cross-validation study on the entire data set of HP and non-HP species and compare the performance of PaPrBaG with that of the other methods tested.

### Classifier training and performance

The results presented below could not be notably improved by further parameter tuning or feature selection efforts. An auxiliary assessment further shows that our classifiers are very robust (see Supplementary Fig. S1). Across all cross-validation folds, the out-of-bag training error is 0.24 and the error on the (imbalanced) test data set is 0.22. Further, the area-under-curve (AUC) is 0.84 and 0.79 for the training and test reads, respectively. Hence, predictor performance generalises well to independent data. The degree of certainty of a read prediction can be measured by the prediction probability (see Methods). We observe that certainty increases continuously from noisy predictions at probabilities around 0.5 to very accurate predictions at probabilities close to 0 or 1. This confirms that prediction probability is indeed related to prediction certainty.

### Predictions for individual reads

**Figure 2:**
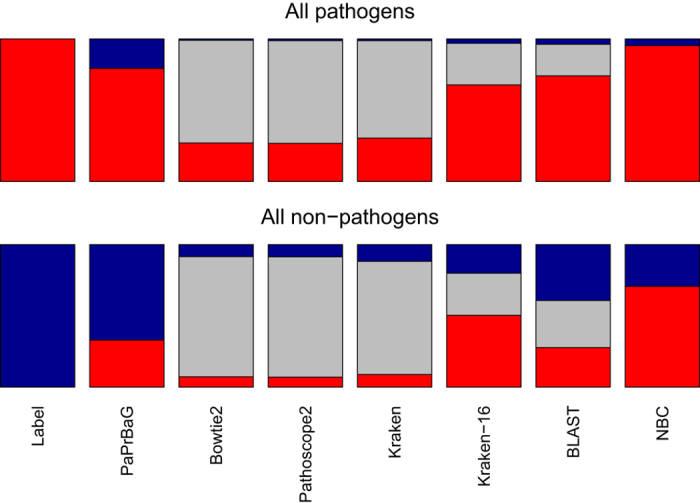
Read predictions for all HPs (top) and non-HPs (bottom). Each bar shows the number of reads predicted to be of HP (red), non-HP (blue) or unknown (gray) origin. The left-most bars show the ground truth. Strikingly, Bowtie2, Pathoscope2 and Kraken fail to classify the majority of the reads. Kraken-16 and BLAST still miss a considerable fraction of reads whereas the machine learning based approaches always return a prediction. All methods show true and false predictions to a varying extent. While PaPrBaG shows similar errors for both HPs and non-HPs, all other methods suffer from a substantial bias. Few reads from HPs are falsely classified as non-HPs. Conversely, for non-HPs, the number of falsely classified reads is similar to or even exceeds the number of correctly classified reads.

Each method initially provides predictions for all individual test reads. In PaPrBaG, the majority of trees either votes for an HP or non-HP origin of a given read. For the other tools, the prediction is either a match to an HP, a non-HP or no match at all. An overview of the per-read results is given in Fig. 2. PaPrBaG always makes a prediction, yet many of them are false positive or false negative. Bowtie2, Pathoscope2 and Kraken can only make predictions for a minority of all reads. Kraken-16 and BLAST are able to map the majority of reads, but still leave a considerable fraction unmapped. All other methods also yield false predictions, particularly false positive ones. As the bottom plot reveals, their false positive rates are similar or even higher than the corresponding fractions of true negatives. This problem reflects the imbalance in the training data, which only PaPrBaG addresses explicitly, by design (see subsection *Benchmark*).

### Phenotype prediction by majority vote

Due to the generally high number of false predictions (see Fig. 2), a single read matching to an HP is generally not sufficient for reliable phenotype prediction. An elementary prediction metric was introduced by the *majority prediction rule* (see Methods). It compares the amount of read evidence for the presence of an HP and a non-HP and assigns the better-supported phenotype.

The classification results for all genomes are shown in Table 1. Most organisms are classified correctly by all methods, with accuracy values ranging from 0.88 to 0.93. This demonstrates that it is indeed possible to infer the pathogenicity phenotype of a novel species solely based on sequencing data. Further, the different methods show different degrees of specificity and sensitivity. Since the test data set is highly imbalanced, however, the *Matthews Correlation Coefficient* (MCC) is more appropriate to compare the performance between different methods. As Table 1 shows, Kraken performs best, followed by Pathoscope2, Bowtie2, BLAST and PaPrBaG. Kraken-16 and NBC yield strongly biased predictions and have a lower MCC. We additionally evaluated the performance of Bowtie2 and Pathoscope2 with a larger reference database containing all strains of all training species. Here, the classifications become more sensitive and less specific, which reflects the larger bias towards HPs in the training set. Overall, the effect of the larger database is small. We further assessed the performance of all approaches in a ROC analysis. Here, instead of the majority, a varying decision threshold determines phenotype prediction. As Supplementary Fig. S2 reveals, BLAST, NBC and PaPrBaG yield the highest area-under-curve (AUC), whereas Kraken, Pathoscope and Bowtie perform worst. Note however that the decision threshold is not known a priori, which fundamentally limits the use of the AUC in practice.

Note that, in this classification scheme, the uncertainty originating from the large number of unmapped reads is ignored. Hence, conclusions are drawn based on the information from an average of 6% (Bowtie2, Pathoscope2), 14% (Kraken) and 78% (BLAST) of all available reads. For Bowtie2 and Pathoscope2, less than 100 reads got mapped for 89 test species, and in two cases not a single read was mapped. Conversely, PaPrBaG and NBC provide predictions for all reads and species. We discuss a different performance metric that explicitly considers the unmapped reads in the *minimum detection threshold* evaluation below.

### Prediction stability with increasing taxonomic complexity

**Figure 3:**
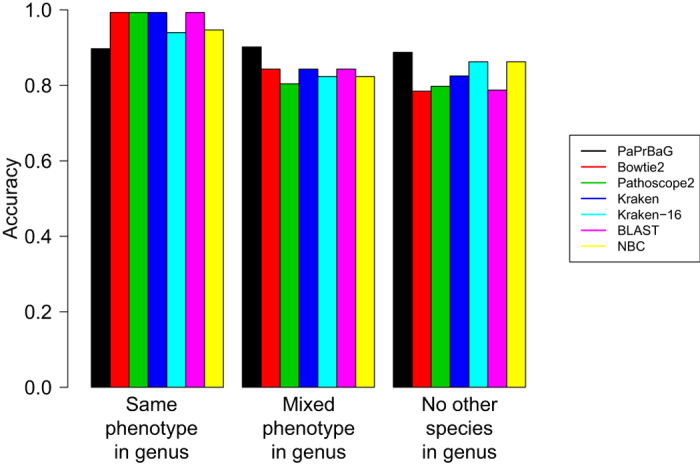
Performance effects of taxonomic complexity. This re-analyses method performance as presented in Table 1 by taking into account the complexity of the respective taxonomic environment. The first set of columns represents those test genomes with one or more training genomes from the same genus that all show a matching phenotype (291 cases), the second set those with at least one training genome for each phenotype from the same genus (51 cases), and the rightmost set those with no other member of the same genus in the training set (80 cases). PaPrBaG maintains stable accuracy in all three settings, clearly outperforming the other approaches when closely related species have different phenotypes or no closely related species exist at all.

Figure 3 re-analyses the results presented in Table 1 in light of the different ‘taxonomic neighborhood’ of each test genome. When all species of the same genus in the training database have the same phenotype as the test genome, other approaches tend to be more accurate than PaPrBaG. Conversely, when confronted with a more complex environment–either when closely related species have different labels or no closely related species exist at all–PaPrBaG is more accurate. Remarkably, the method maintains similar accuracy across all neighborhood types. These findings indicate that PaPrBaG–unlike the other approaches–picks up on a subtle phenotypic signal beyond the overall strong phylogenetic (distance) one.

### Consensus filter

The heterogeneous prediction results of the individual classifiers suggested it might be worthwhile to combine them. Accordingly, we introduced a *consensus filter* (see Methods). It filters and evaluates predictions that coincide between different classifiers. The lower part of Table 1 shows the performance of selected combinations of methods. Combining PaPrBaG with either Bowtie2, Pathscope2, Kraken or BLAST increases performance substantially when compared with any of the individual methods. We find accuracy values above 0.95 and MCC values above 0.7. Combining PaPrBaG with Bowtie2 and Kraken achieves the highest performance, closely followed by PaPrBaG with either Kraken or Pathoscope. The single best combination without PaPrBaG, Pathoscope2 + Kraken, yields good results, but is outperformed by the combinations including PaPrBaG. The combination Pathoscope2 + NBC has a lower MCC than Pathoscope2 alone. Other combinations of Bowtie2, Pathoscope2, Kraken, BLAST and NBC without PaPrBaG showed no substantial improvements and have been omitted from Table 1. As the read mapping tools make highly overlapping predictions, they also tend to make the same errors. Conversely, PaPrBaG behaves differently and makes unique predictions. In conclusion, although combining two or more classifiers does not increase the overall performance, it increases prediction confidence for a subset of the data. Therefore, it is favourable to use different classification tools when maximum confidence is desired.

### Coverage dependency

**Figure 4:**
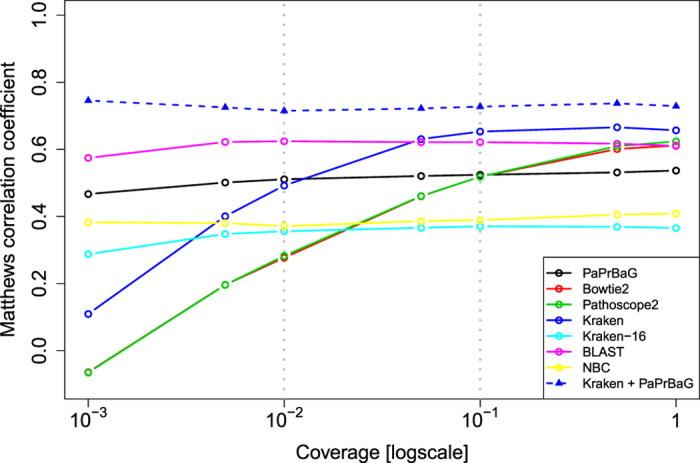
Classification performance for different genome coverages. As coverage decreases, so does the performance of Bowtie2, Pathoscope2 and Kraken. Conversely, BLAST and PaPrBaG still deliver sound results at coverages as low as 0.001. The triangles show results for the consensus filter when combining PaPrBaG and Kraken. It achieves high performances at all coverage levels, however, at the cost of filtering out more and more data.

The results discussed so far were based on test genomes sequenced with a coverage of 1. However, in an experimental situation the coverage may be well below 1, in particular for metagenomic data. In the following, we elucidate how classification performance depends on the coverage of the test genomes. Since fluctuations may play a more important role for low coverages, we averaged over 100 simulation repeats. The corresponding results are shown in Fig. 4. The performance of BLAST and PaPrBaG is rather stable over the entire range of coverages. Both are still reasonably sensitive even at extremely low coverages of about 0.001. The same holds for Kraken-16 and NBC albeit with a lower MCC across all coverages. Kraken’s performance substantially decreases for coverages lower than 0.05. Bowtie2 and Pathoscope2 only work well for high coverages; below 0.1, their performance decreases rapidly. As discussed above, for Kraken, Bowtie2 and Pathoscope2 only a small amount of reads can be mapped at all at a coverage of 1. Hence, a reduction of the number of reads means that it becomes more and more likely that no read can be mapped to the reference at all. Consequently, their performance drops to the noise level. Also shown are the results obtained after applying the *consensus filter*. Combining Kraken and PaPrBaG leads to confident predictions at all coverage levels.

### Prediction certainty

**Figure 5:**
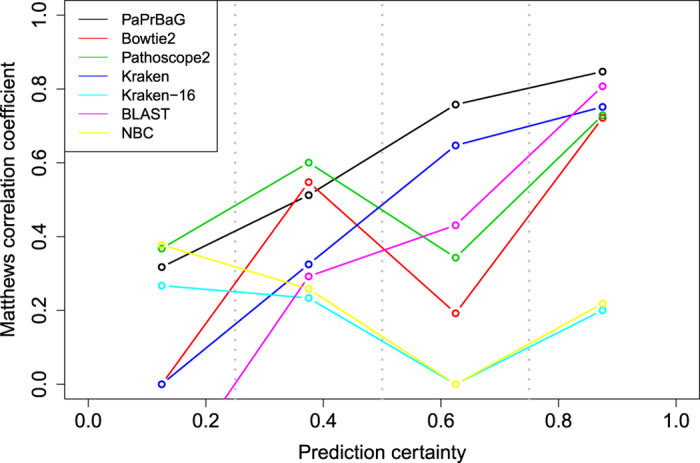
Fidelity of prediction certainty. Each prediction is associated with uncertainty. Here, we pooled predictions within each certainty interval and measured the prediction performance (MCC). PaPrBaG, Kraken and BLAST show a steady increase in performance with increasing certainty. PaPrBaG achieves the highest MCC among all methods compared.

Each prediction made using the *majority prediction rule* is associated with a certain confidence. We can quantify this as *prediction certainty*, as explained in the section Methods. Figure 5 shows the performance of the different classifiers at different certainty levels. It reveals that the performances of PaPrBaG, Kraken and BLAST increase strongly with prediction certainty. Predictions of PaPrBaG with certainty values between 0.75 and 1 achieve the highest MCC of 0.85. Note moreover that for the other approaches the prediction certainty is almost always found at high values. Thus, for these approaches there is a smaller performance gain when comparing very certain to average predictions. Hence, we can conclude that a high prediction certainty is related to a particularly high prediction performance for PaPrBaG.

### Minimum detection threshold

**Figure 6:**
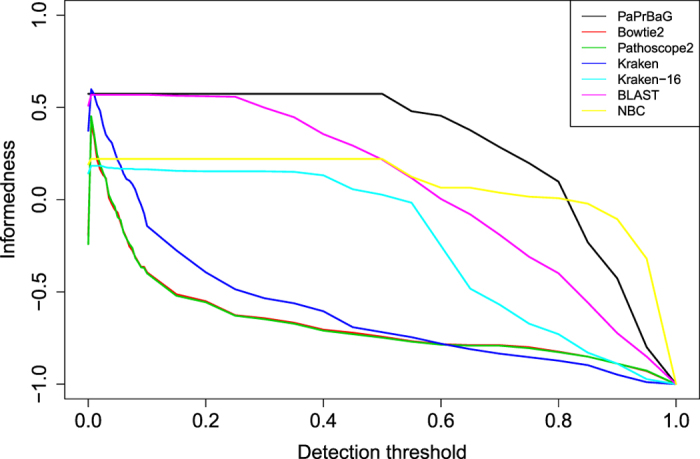
Classification with minimum detection threshold. Predictions are only made for genomes where the read evidence supporting a phenotype exceeds the detection threshold (given relative to the total number of reads). Initially, most approaches show high informedness, which is a joint measure of sensitivity and specificity defined as *I* = TPR + TNR − 1. As the detection threshold is increased above 0.1, Bowtie2, Pathoscope and Kraken yield insufficient numbers of reads with phenotype evidence and they are no longer informative. Only PaPrBaG and BLAST show an informedness above 0.5. For most values of the detection threshold, PaPrBaG exhibits the highest informedness.

The performance evaluations above were based on the *majority prediction rule*. There, the overall prediction is determined by the majority of the individual read predictions. However, the decision basis may occasionally be very narrow: for the read mappers only a few hundred reads (a few percent of all reads) may map to any of the reference genomes. In these cases e.g. a small number of contaminant reads may falsify the prediction result. Therefore, we further studied the effect of varying the *minimum detection threshold*. Generally, choosing a higher threshold should lead to increased prediction confidence. Figure 6 summarises the results in terms of informedness. For low detection thresholds most methods attain high sensitivity and specificity, and therefore high informedness. However, for detection thresholds around 0.1, requiring 10% of all reads to support a phenotype, only PaPrBaG and BLAST reach an informedness above 0.5. Conversely, the informedness of Bowtie2, Pathoscope2 and Kraken drops below 0, i.e. their predictions are at noise level. Increasing the detection threshold further, fewer and fewer predictions can be made and eventually the informedness of all methods approaches −1. However, at most threshold levels, PaPrBaG shows the highest informedness. Hence, when it is desired that a high number of reads support a phenotype, PaPrBaG is the most informative method.

### Run time comparison

Table 2 lists the median run times for a complete genome prediction, respectively. While prediction with PaPrBaG is relatively fast, feature extraction and loading the trained random forest consumes a considerable amount of time. The fastest method is read mapping with Bowtie2. However, post-processing the mapped reads takes time. Note that this step is not part of Bowtie2 itself and hence has not been optimised for speed. Pathoscope2 additionally filters the read mapping results, which leads to faster post-processing. Mapping reads to the larger reference database containing all training strains increases the run times considerably. Prediction with Kraken takes unexpectedly long. It benefits from its high speed only for larger read sets. Note that pre-processing here includes the time-consuming step of loading the Kraken database, as well as the relatively slow translation module. Finally, BLAST is relatively slow, and NBC is the slowest method by far.

### Metagenomic example

PaPrBaG can be used for metagenomic analysis, particularly in combination with other fast methods such as Bowtie2 or Kraken. In this, the role of the latter is to quickly handle all reads related to known genomes, while PaPrBaG serves to assess the pathogenic potential of those reads that cannot be mapped, stemming from highly divergent strains and novel species. To illustrate this, we devised a specific example. This concerns the metagenome of a clinical sample consisting of a number of highly-abundant, known non-HPs and a single, underrepresented and unknown HP species (for details see section *Methods* and Table 3). Note that real clinical samples will often be contaminated with reads from the human host, which may be initially subtracted using specialised tools^18^,^51^.

In our example (see Table 3), reads originating from the known non-HPs can be mapped with very high accuracy (>99%). Conversely, Bowtie2 and Kraken cannot map the vast majority (99 and 98%) of the unknown-HP reads, with the few remaining ones mapping to 6 and 23 different species, respectively. The false positive hits outnumber the true positives and also the number of false negatives is relatively high. Hence, the mapping-based evidence for a pathogen is scarce - below the noise level as marked by the false positive rate. This situation changes entirely when PaPrBaG is used on the remaining, unmapped reads. 5,614 (85%) of the reads not mapped by Bowtie2 show HP evidence (see also Supplementary Fig. S3). Similarly, 5,494 reads of the Kraken remainder support the HP hypothesis. As an alternative to PaPrBaG, BLAST may be used to classify the unmapped reads. However, BLAST can align only about half of the reads in both cases. More importantly, only 9% support the HP hypothesis. Further, strikingly, BLAST aligns to 192 different species.

Altogether, these findings demonstrate the relevance of PaPrBaG in the metagenomic context. In particular, it can shed light on clinical samples containing a high number of unmapped reads. The example also further stresses the value of PaPrBaG in combination, rather than competition, with other methods. While the discussed example has been chosen for illustrative purposes, it is still highly representative. *Gardnerella vaginalis* is a species with no other representatives in the same genus. As discussed above, PaPrBaG is generally superior to the other approaches in this scenario. Further, as noted above, Bowtie2 and Kraken cannot map a large fraction of reads for most of the genomes. Additionally, it should be clear that in case that the experimental condition provides less reads for the pathogen, mapping will fail more frequently (see subsection *Coverage dependency* and Fig. 4).

## Discussion

In this contribution, we investigated the potential of predicting the pathogenicity phenotype of novel bacterial species directly from sequencing reads. To that end, we developed a machine learning method that combines feature extraction with random forest prediction, PaPrBaG. We further compiled a new data set of bacterial genomes with reliable pathogenicity information as inferred using a rule-based protocol. PaPrBaG and several other alignment- and composition-based approaches were extensively tested on this data.

It is notable that all methods achieved high accuracy for the difficult task of novel species classification. Remarkably, PaPrBaG was one of the few tools achieving solid predictions across a wide range of coverages. In contrast to most approaches, it produced reliable predictions for genomic coverages as low as 0.001. At high coverages, PaPrBaG performed competitively and, in particular, better than composition-based approaches.

For reliable pathogen identification it is desirable to obtain relevant information from as many reads as possible. Whereas most methods could match only a small fraction of reads to HPs or non-HPs, PaPrBaG always makes a prediction. This proved to be key when requiring a minimum amount of read evidence for prediction. In this case, PaPrBaG was found to be the most informative approach. The reliability of a prediction is also related to the prediction certainty. Here we could show that when selecting the most certain predictions only, PaPrBaG achieved the best performance of all methods discussed.

It is further interesting that although PaPrBaG was trained on sequencing reads covering only a small fraction of the training genomes, the approach worked strikingly well. Hence, PaPrBaG is able to make solid predictions while it *sees* much less of the training data than the other methods.

Whereas the existing tools are based on taxonomic classification, PaPrBaG is a conceptually novel approach. It is a binary classifier that learns directly from a set of genome sequences of HP and non-HP species. Therefore, it is not surprising that the predictions of PaPrBaG are more diverse when compared with other approaches. In particular, PaPrBaG makes unique true and false predictions, which is beneficial when using a consensus approach. Combining the existing approaches with PaPrBaG led to particular high performances, better than any individual classifier. As we could further show explicitly, only PaPrBaG maintains a high prediction accuracy also when the phylogenetic signal is weak. Thus, as opposed to mere taxonomic classification, PaPrBaG can learn phenotype-related signals.

In terms of run times, PaPrBaG is much faster than BLAST and NBC, whereas it is not optimised for speed like Bowtie2 and Kraken. Nevertheless, the pure prediction times are competitive with these methods. As has been shown, considering a larger reference database leads to significantly longer prediction times when using competing approaches. In contrast, while PaPrBaG’s main bottleneck is extracting the read features, the run time of this step is independent from the initial database size. In addition, feature extraction could potentially be optimised further, e.g. by considering genomic features only. Finally, any speed loss due to increasing forest complexity (e.g. due to more diverse training data becoming available and, potentially, increased sampling of training reads) could be remedied via tree pruning. Thus, PaPrBaG provides a scalable solution for even larger data set sizes.

Apart from the application of PaPrBaG on single-genome read sets, we could also demonstrate its potential in a metagenomic context. Here, its solid performance at very low coverages and its capability to pick up phenotype-related signals may prove highly valuable. Our practical example emphasised that the combined use of mapping-based approaches and PaPrBaG is a promising strategy to quickly and reliably detect bacterial pathogens in clinical samples. Additionally, PaPrBaG’s ability to score each read for its pathogenic potential allows for the simple prioritisation of reads for further downstream analysis.

This work strongly depends on reliable phenotype information. We introduced a strategy within PaPrBaG to overcome the pathogen bias. Nevertheless, PaPrBaG and other methods have higher sensitivity than specificity, and it would be interesting to see how the methods would work with higher numbers of labelled bacterial species, particularly non-HPs. Moreover, we expect the newly created data set to stimulate further developments in the pathogenicity prediction field.

Finally, it is worth mentioning that the approach pursued by PaPrBaG is not restricted to the classification of the complex phenotype *pathogenicity*. It is rather a general workflow for the classification of labelled genomes, and potential further applications range from bacterial host and habitat prediction, taxonomic classification to human and microbial read separation.

## Additional Information

**How to cite this article**: Deneke, C. *et al*. PaPrBaG: A machine learning approach for the detection of novel pathogens from NGS data. *Sci. Rep.* **7**, 39194; doi: 10.1038/srep39194 (2017).

**Publisher's note:** Springer Nature remains neutral with regard to jurisdictional claims in published maps and institutional affiliations.

## Supplementary Material

Supplementary Figures and Tables

## Figures and Tables

**Table 1:** Prediction results for majority prediction rule.

	TPR	TNR	ACC	F1	MCC
PaPrBaG	0.91	0.70	0.88	0.93	0.54
Bowtie2	0.95	0.66	0.91	0.95	0.61
Pathoscope2	0.94	**0.72**	0.91	0.95	0.62
Kraken	0.97	0.64	**0.93**	**0.96**	**0.66**
Kraken-16	**0.99**	0.19	0.89	0.94	0.37
BLAST	0.96	0.60	0.92	0.95	0.61
Bowtie2_All Strains_	0.96	0.60	0.91	0.95	0.58
Pathoscope2_All Strains_	0.96	0.66	0.92	0.95	0.63
NBC	0.99	0.23	0.90	0.94	0.41
Bowtie2 + PaPrBaG	0.97	0.77	0.95	0.97	0.71
Pathoscope2 + PaPrBaG	0.97	**0.81**	0.95	0.97	0.74
BLAST + PaPrBaG	0.97	0.74	0.95	0.97	0.70
Kraken + PaPrBaG	0.97	0.76	0.95	0.98	0.73
Pathoscope2 + Kraken	0.97	0.69	0.94	0.97	0.70
Pathoscope2 + NBC	**0.99**	0.44	0.95	0.98	0.60
Bowtie2 + Kraken + PaPrBaG	0.98	0.78	**0.96**	**0.98**	**0.75**

The first set of entries shows the performance of the individual methods. Bowtie *All Strains* and Pathoscope *All Strains* represent a variation where the reference data set contains all strains of a species in the training set. Below the horizontal line, we show results for the combination of methods with the *consensus filter*. In these cases, the performance is given for those genomes that have predictions agreeing between two or more individual classifiers. Overall, combining PaPrBaG with Bowtie2 and Kraken yields the best performance. (TPR = True positive rate, TNR = True negative rate, ACC = Accuracy, F1 = F1-score, MCC = Matthews Correlation Coefficient).

**Table 2:** Comparison of run times.

Method	Pre-processing	Prediction	Post-processing
PaPrBaG	180	29	0
Bowtie2	0	14	78
Pathoscope2	0	15	2
Bowtie2_All Strains_	0	165	105
Pathoscope2_All Strains_	0	169	12
Kraken	990	62	33
Kraken-16	1,833	18	48
BLAST	0	498	1
NBC	0	13,901	311

All tools except NBC were run in single-threaded mode on an SMP machine with 48 cores and 256 GB RAM. Given are the median times (in seconds) for a complete genome prediction as well as for the required pre- and post-processing steps. Bowtie2 and Pathoscope2 are the fastest methods, followed by Kraken and PaPrBaG. Note that Kraken takes particularly long to load its database.

**Table 3:** Summary of metagenomic example.

	Ground truth	Bowtie	Kraken
*Lactobacillus salivarius*	200,000	199,982	199,915
*Lactobacillus reuteri*	200,000	199,788	199,763
*Lactobacillus rhamnosus*	200,000	200,000	199,963
*Bifidobacterium breve*	200,000	199,832	199,641
*Haemophilus parainfluenzae*	200,000	199,861	199,648
*Gardnerella vaginalis*	6,604	0	0
Other HP	0	271	338
Other non-HP	0	278	51
Matched to higher taxonomy	—	0	828
Unmatched	—	6,592	6,457
True Positives (TP)	—	6	34
False Positives (FP)	—	265	304
False Negatives (FN)	—	6	70
TP PaPrBaG	—	5,614	5,494
FP PaPrBaG	—	978	963
TP BLAST	—	605	589
FP BLAST	—	2,927	2,809
Unmatched BLAST	—	3,060	3,059

Shown is the true read composition as chosen for this example as well as the composition predicted by Bowtie2 or Kraken (top panel). Note that in both cases, the vast majority of the HP reads cannot be mapped. Furthermore, although the non-HPs can be mapped with high accuracy, the false positives outnumber the true positives (middle panel). Also the number of false negatives is relatively high. Hence, after read mapping the presence of a pathogen remains uncertain. Whereas PaPrBaG makes correct predictions for more than 85% of the remaining, unmapped reads (lower panel), BLAST can only match half of those with a (usually non-HP i.e. misleading) reference genome.
