# Supplementary material for: PaPrBaG: A machine learning approach for the detection of novel pathogens from NGS data
Source: Sci Rep. 2017 Jan 4;7:39194. doi: 10.1038/srep39194 (PMC5209729; doi:10.1038/srep39194)
Supplement: Supplementary Figures and Tables [file srep39194-s1.pdf]

PaPrBaG: A machine learning approach for the detection of novel pathogens from NGS data

Carlus Deneke[+], Robert Rentzsch[+], and Bernhard Y. Renard[+,*]

[+]Research Group Bioinformatics (NG4), Robert Koch Institute, 13353 Berlin, Germany.
[*]renardb@rki.de

Supplementary Figures and Tables

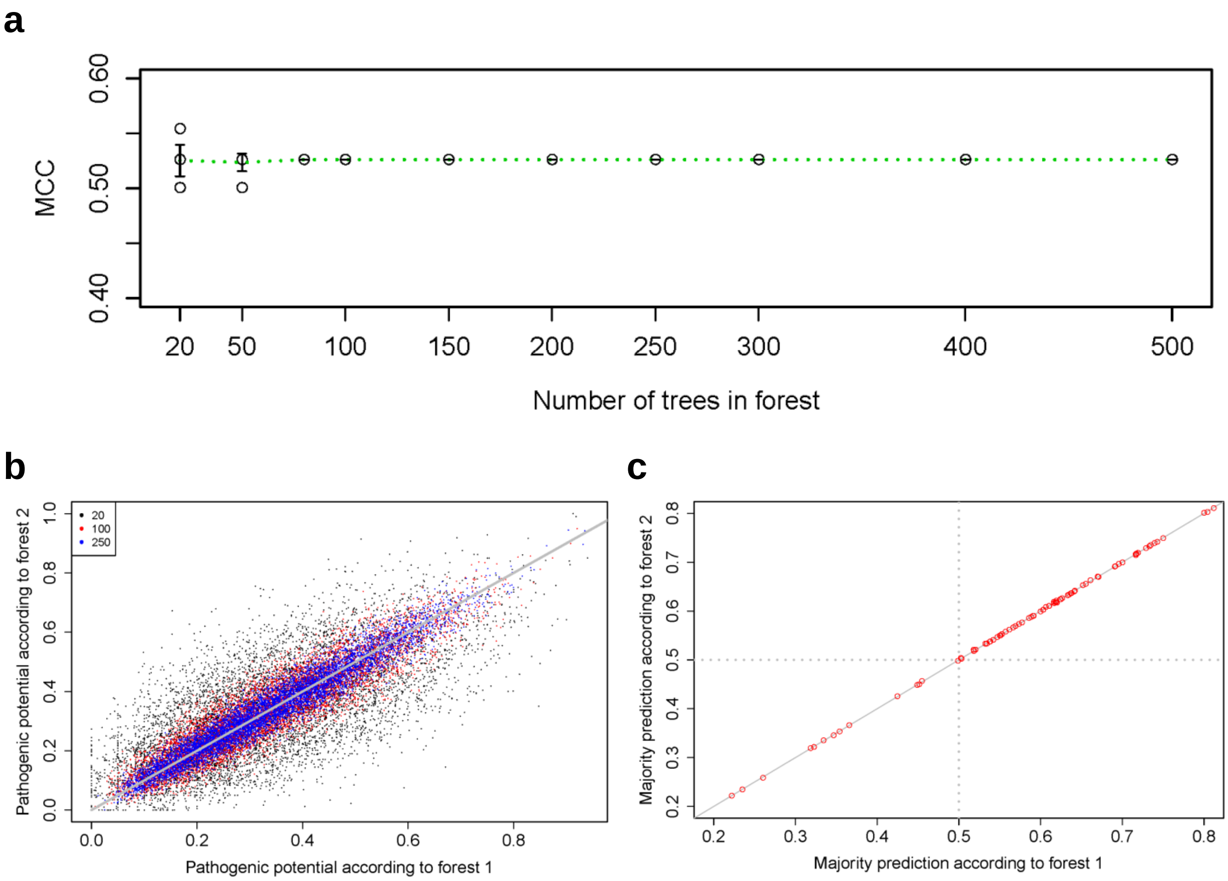

Supplementary Figure S1: **Tree number dependence and robustness of classifier**. Panel (a) shows the Matthews-Correlation-Coefficient (MCC) of the classification of 84 genomes (pertaining to one training fold) for a wide range of trees in a forest. Circles denote different replica runs, the corresponding standard deviations for each given tree number are indicated by the black bars and the green dotted line shows the mean for a given tree number. Fluctuations play some role for a small number of trees between 20 and 50 per forest. For 80 or more trees the results are stable with no difference between different replica and increasing tree number. Panel (b) compares the read-based performance for one test genome (PRJNA89653, [Eubacterium] nodatum) for two replica classifiers. Note that the noise is relatively high when forests consist of 20 trees only (black dots). Conversely forests with 100 (red) and 250 (blue) trees tend to yield very similar predictions for each read in the sample. The inherent noise of a random forest classifier vanishes when comparing genome predictions of two replica classifiers. Both forests yield nearly the identical mean pathogenic potential per genome (red circles, shown for forests with 100 trees each). In conclusion, these results indicate that the random forest classifier used in this setting is very robust and sufficiently trained with 100 trees per forest.

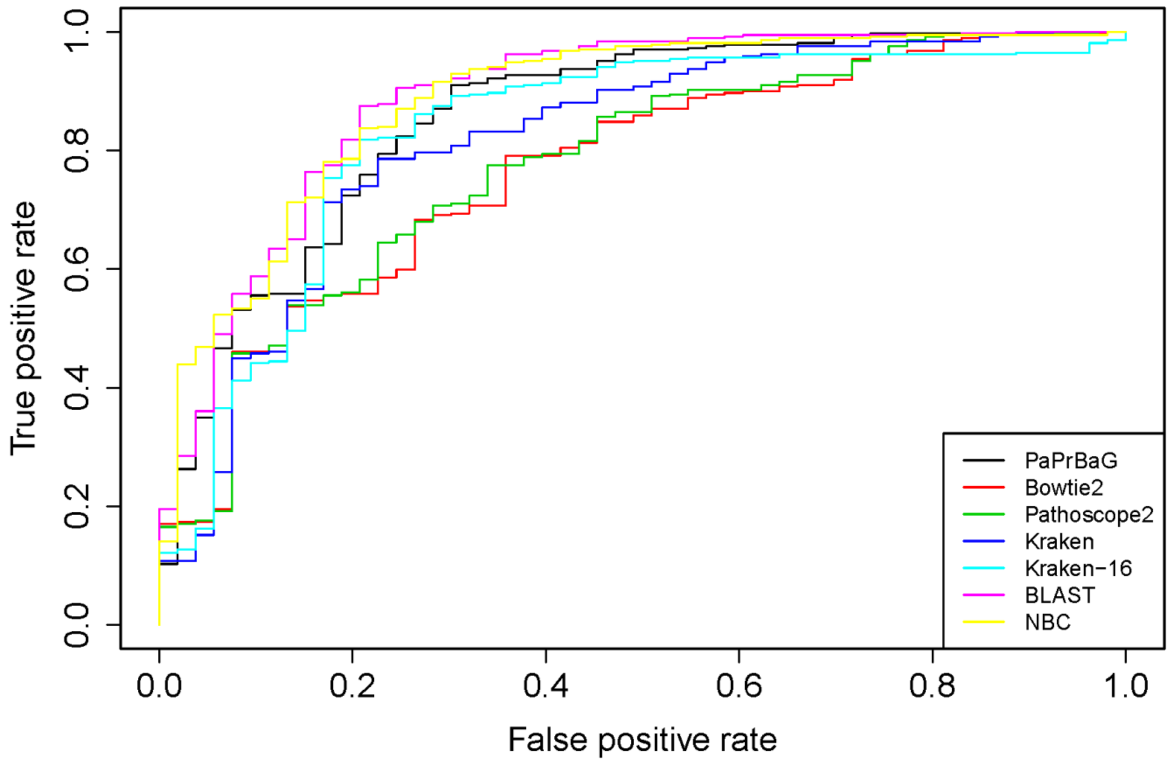

Supplementary Figure S2: ROC curve for different prediction methods. The receiver-operating characteristic is obtained by continuously varying the decision threshold, i.e. instead of the majority prediction a tuneable parameter classifies predictions as either pathogenic or non-pathogenic. As the curves reveal, BLAST, NBC and PaPrBaG yield the best performances with area-under-curve (AUC) of 0.89, 0.88 and 0.86, respectively. In contrast to the majority prediction rule, Kraken, Pathoscope2 and Bowtie2 perform worse (AUC 0.82, 0.78 and 0.77, respectively). Kraken-16 performs intermediate with an AUC of 0.83. In summary, the ROC analysis sheds a different light on the results discussed in Table 1 (see main text). However, its use in practical application is limited since in an experiment the optimal threshold is not known a-priori. Hence, rather than giving a concrete prediction, the ROC yields the statistical distinguishability between the two phenotypes.

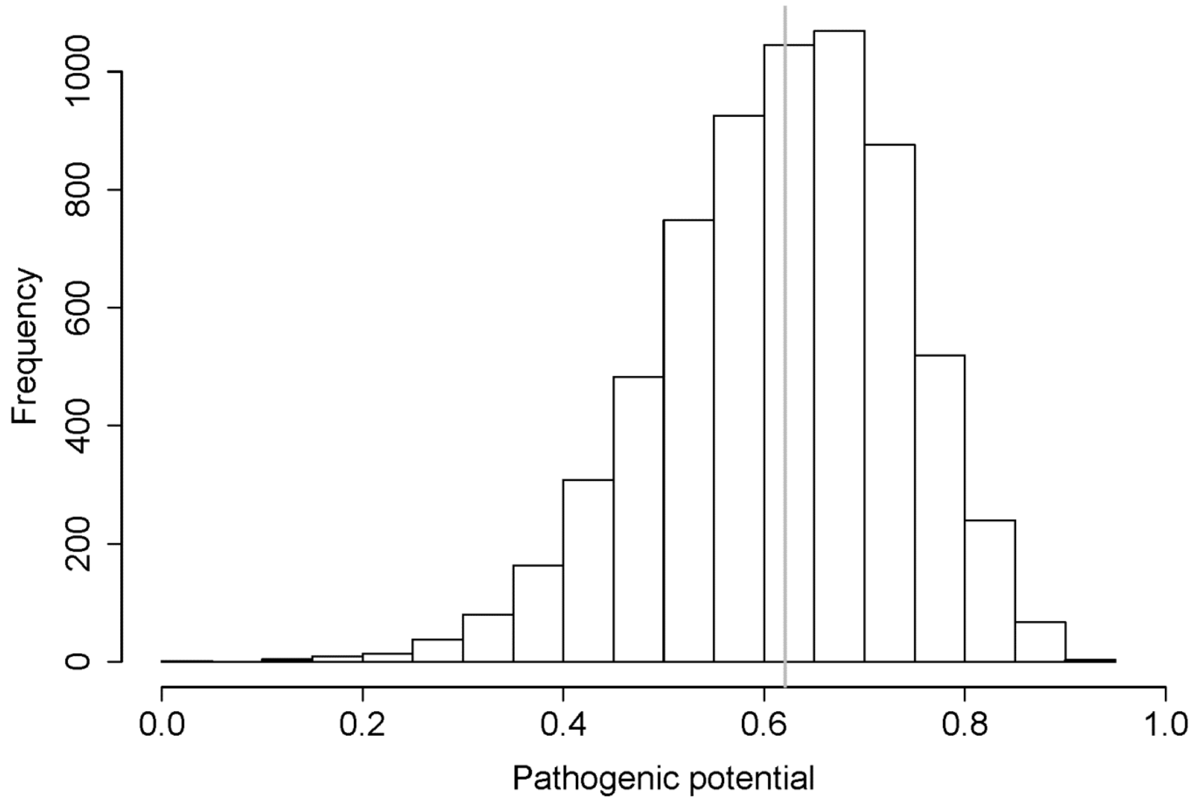

Supplementary Figure S3: Pathogenic potential of unmapped reads in metagenomic sample. Shown are all reads that were not mapped by Bowtie2. The majority of the reads (> 85 %) have a pathogenic potential larger than 0.5 and the mean prediction is 0.62 (grey line). Thus PaPrBaG provides a strong signal for the presence of a pathogen.

Supplementary Table S1: The top 100 most important features. Features are ranked by permutation importance and results are shown for the first fold of the five-fold cross-validation (with very similar results for the other folds). The four amino acid index (see references 41-43 for details) accessions occurring are (i) The Kerr effect of amino acids in water: The Kerr-constant increments [KHAG800101], (ii) Characterization of multiple bends in proteins: Normalized relative frequency of double bend [ISOY800107], (iii) Shape and surface features of globular proteins: Correlation coefficient in regression analysis [PRAM820103] and (iv) Protein secondary structure: Normalized frequency of beta-sheet in alpha+beta class [PALJ810111]. Furthermore, all k-mer features are listed including (and consider) the respective reverse complement.

Rank	Feature name	Permutation importance	Gini importance
1	Nucleotide 3-mer relative frequency: GGA,TCC	0.01071	11608.1
2	Nucleotide 3-mer relative frequency: CGC,GCG	0.00775	4989
3	Nucleotide 3-mer relative frequency: CCG,CGG	0.0061	3672.5
4	Nucleotide 3-mer relative frequency: ATA,TAT	0.0059	3136.5
5	Relative frequency of base pair: A,T	0.00574	1953.9
6	Relative frequency of base pair: C,G	0.00554	3827.9
7	Nucleotide 3-mer relative frequency: CGA,TCG	0.00511	3370
8	Nucleotide 2-mer relative frequency: GC,GC	0.00506	3574.9
9	Nucleotide 2-mer relative frequency: CG,CG	0.00496	3583
10	Nucleotide 4-mer relative frequency: CGGA,TCCG	0.00491	4118.9
11	Nucleotide 4-mer relative frequency: CCGG,CCGG	0.0048	4495.5
12	Nucleotide 2-mer relative frequency: AA,TT	0.0041	2233.2
13	Nucleotide 4-mer relative frequency: GCGC,GCGC	0.00407	1936.6
14	Nucleotide 3-mer relative frequency: CTA,TAG	0.00385	3268
15	Nucleotide 4-mer relative frequency: CTAA,TTAG	0.00344	3167.7
16	Nucleotide 2-mer relative frequency: AT,AT	0.00336	1802.1
17	Nucleotide 4-mer relative frequency: GGAA,TTCC	0.00323	3300.7
18	Nucleotide 3-mer relative frequency: AAA,TTT	0.00304	1661.5
19	Nucleotide 4-mer relative frequency: CGCG,CGCG	0.00287	2316.4
20	Nucleotide 4-mer relative frequency: GCGA,TCGC	0.00283	2975.4
21	Nucleotide 3-mer relative frequency: TAA,TTA	0.00275	1794.2
22	Spaced word count: CGCG,CGCG	0.00266	1752.9
23	Nucleotide 4-mer relative frequency: CATA,TATG	0.00254	2392.6
24	Spaced word count: CCCG,CGGG	0.00254	1473.8
25	Spaced word count: CGAG,CTCG	0.00253	1789.4
26	Nucleotide 3-mer relative frequency: GAA,TTC	0.00252	3029.3
27	Nucleotide 4-mer relative frequency: CGAC,GTCG	0.00252	1510.5
28	Spaced word count: GCGA,TCGC	0.00244	1815.9
29	Nucleotide 3-mer relative frequency: AGA,TCT	0.00235	1486.6
30	Spaced word count: GCGC,GCGC	0.00226	1368.2
31	Nucleotide 2-mer relative frequency: GA,TC	0.00226	2222.6
32	Nucleotide 2-mer relative frequency: TA,TA	0.00219	1495.9
33	Nucleotide 4-mer relative frequency: CTAG,CTAG	0.00215	2023
34	Nucleotide 4-mer relative frequency: ATAT,ATAT	0.00213	1348.7
35	Spaced word count: CGCC,GGCG	0.00208	1879.6
36	Nucleotide 4-mer relative frequency: CTCC,GGAG	0.00206	1971.5
37	Spaced word count: AGCG,CGCT	0.00195	1652.5
38	Nucleotide 3-mer relative frequency: CAG,CTG	0.00195	1572.2
39	Nucleotide 4-mer relative frequency: ATAC,GTAT	0.00191	1659.9
40	Nucleotide 3-mer relative frequency: ATG,CAT	0.00184	1964.9
41	Spaced word count: GCCC,GGGC	0.00178	1008.6
42	Nucleotide 4-mer relative frequency: CGCC,GGCG	0.00174	1323.3
43	Nucleotide 4-mer relative frequency: CAGA,TCTG	0.00174	1890.9
44	Spaced word count: AAAA,TTTT	0.00171	1384.5
45	Nucleotide 3-mer relative frequency: GCC,GGC	0.00171	1282.1
46	Spaced word count: GAGC,GCTC	0.0017	1935.3
47	Nucleotide 2-mer relative frequency: CC,GG	0.00169	1206.1
48	Spaced word count: CGGC,GCCG	0.00168	1586.8
49	Nucleotide 3-mer relative frequency: ACA,TGT	0.00166	1807.7
50	Nucleotide 4-mer relative frequency: AAAG,CTTT	0.00162	932.8
51	Spaced word count: AAAG,CTTT	0.00157	1438.2
52	Spaced word count: GCTA,TAGC	0.00156	1745.1

**53**	Nucleotide 4-mer relative frequency: TAAA,TTTA	0.00154	1251.2
**54**	Nucleotide 4-mer relative frequency: TATA,TATA	0.00153	1166.8
**55**	Spaced word count: CGAC,GTCG	0.0015	883.8
**56**	Nucleotide 4-mer relative frequency: AAAA,TTTT	0.00149	1173.4
**57**	Nucleotide 4-mer relative frequency: TTAA,TTAA	0.00137	1595.1
**58**	Nucleotide 4-mer relative frequency: ACAG,CTGT	0.00135	1608.6
**59**	Spaced word count: TACA,TGTA	0.00135	1234.9
**60**	Spaced word count: AAAT,ATTT	0.00133	830.4
**61**	Spaced word count: ATAT,ATAT	0.00131	1116.4
**62**	Spaced word count: CATA,TATG	0.00131	1243.4
**63**	Spaced word count: CCGC,GCGG	0.00129	1166
**64**	Spaced word count: ATAA,TTAT	0.00128	1019.3
**65**	Spaced word count: AAGA,TCTT	0.00125	1230.8
**66**	Spaced word count: ATAC,GTAT	0.00124	1051.5
**67**	Nucleotide 2-mer relative frequency: CA,TG	0.00124	1509.8
**68**	Amino acid index score: KHAG800101	0.00121	1377.8
**69**	Spaced word count: AGAA,TTCT	0.00117	1287.9
**70**	Nucleotide 3-mer relative frequency: AAT,ATT	0.00117	932.9
**71**	Amino acid index score: ISOY800107	0.00117	1183
**72**	Spaced word count: ACAT,ATGT	0.00116	1367.6
**73**	Spaced word count: GAAA,TTTC	0.00115	1208.2
**74**	Spaced word count: CGTC,GACG	0.00112	1127.7
**75**	Nucleotide 3-mer relative frequency: ACC,GGT	0.00112	1217.2
**76**	Nucleotide 4-mer relative frequency: CGGC,GCCG	0.00111	826.8
**77**	Spaced word count: CAGA,TCTG	0.00111	1692.9
**78**	Spaced word count: ACCG,CGGT	0.00108	1031.6
**79**	Nucleotide 4-mer relative frequency: ACGG,CCGT	0.00107	1455.2
**80**	Amino acid index score: PRAM820103	0.00105	1254.3
**81**	Nucleotide 3-mer relative frequency: AGC,GCT	0.00105	1275.8
**82**	Nucleotide 4-mer relative frequency: AGCG,CGCT	0.00104	1204.3
**83**	Spaced word count: TAAA,TTTA	0.00103	902
**84**	Spaced word count: GACA,TGTC	0.00101	1661
**85**	Nucleotide 3-mer relative frequency: CAA,TTG	0.00101	1272.4
**86**	Nucleotide 4-mer relative frequency: AGGA,TCCT	0.001	1434.7
**87**	Nucleotide 4-mer relative frequency: GCTA,TAGC	0.00099	1109.2
**88**	Nucleotide 4-mer relative frequency: TACA,TGTA	0.00098	1024.4
**89**	Spaced word count: CGGA,TCCG	0.00097	1093.8
**90**	Nucleotide 3-mer relative frequency: GTA,TAC	0.00096	1250.7
**91**	Spaced word count: AGGA,TCCT	0.00094	1559.9
**92**	Amino acid index score: PALJ810111	0.00094	1365.7
**93**	Spaced word count: AATA,TATT	0.00092	742.4
**94**	Nucleotide 4-mer relative frequency: CAAA,TTTG	9.00E-04	788.5
**95**	Nucleotide 3-mer relative frequency: AAG,CTT	9.00E-04	1064.6
**96**	Nucleotide 4-mer relative frequency: ATAA,TTAT	0.00089	694.6
**97**	Spaced word count: CTTC,GAAG	0.00088	1462.3
**98**	Spaced word count: TATA,TATA	0.00088	688.4
**99**	Nucleotide 3-mer relative frequency: CCA,TGG	0.00087	1265.1
**100**	Codon frequency: AGA	0.00085	807.5
